# Hemorrhagic Shock due to Internal Thoracic Artery Injury After Cardiopulmonary Resuscitation

**DOI:** 10.1002/ccr3.72504

**Published:** 2026-04-10

**Authors:** Tatsuya Fujihara, Akiyo Horiguchi, Takahito Taniura, Tetsuro Nikai

**Affiliations:** ^1^ Department of Anesthesiology Shimane University Faculty of Medicine Japan; ^2^ Division of Emergency and Critical Care Department Shimane Prefectural Central Hospital Japan; ^3^ Department of Digestive and General Surgery, Faculty of Medicine Shimane University Shimane Japan

**Keywords:** cardiopulmonary resuscitation, hemorrhagic shock, iatrogenic complication, trans‐arterial embolization

## Abstract

Internal thoracic artery injury can occur after cardiopulmonary resuscitation (CPR). Aggressive fluid resuscitation may mask additional bleeding sites in cases of hemorrhage‐induced cardiac arrest. Persistent hemodynamic instability after return of spontaneous circulation should prompt evaluation for CPR‐related hemorrhagic complications.

## 1 Case Illustrated

**FIGURE 1:**
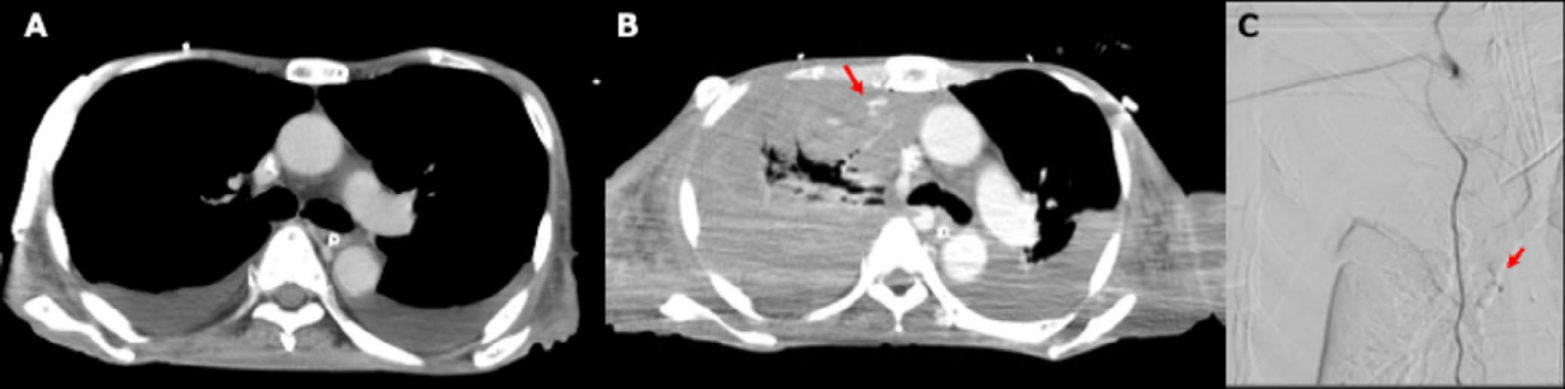
(A) Contrast‐enhanced chest CT obtained immediately after resuscitation from cardiopulmonary arrest showed only small bilateral pleural effusions, with no evidence of extravasation from the internal thoracic artery. (B) Repeat contrast‐enhanced CT performed the following day demonstrated active bleeding from the right internal thoracic artery. (C) Transarterial embolization was performed to control the active bleeding from the right internal thoracic artery.

A 73‐year‐old male patient suddenly underwent cardiac arrest accompanied by a large volume of bloody drainage from a pelvic drain 6 days postoperatively following treatment for a pelvic abscess. Cardiopulmonary resuscitation (CPR) was performed for approximately 6 min, with chest compressions delivered by experienced personnel, including intensive care unit nurses and physicians trained in critical care. Although no mechanical CPR device was used, the team leader provided real‐time feedback on compression quality, including rhythm and depth. No clinical evidence of rib fractures occurred during CPR. Following resuscitation, contrast‐enhanced computed tomography (CECT) revealed active bleeding from the left inferior gluteal artery, and trans‐arterial embolization (TAE) was subsequently performed. Although the patient's vital signs temporarily stabilized after TAE, he required ongoing transfusion and volume resuscitation due to persistent anemia and circulatory instability. Despite aggressive fluid and blood product administration with vasopressor support, the patient remained only partially responsive to resuscitation. On the following day, a chest radiograph showed a reduction in radiolucency in the right lung field. Repeat CECT revealed leakage of the contrast agent from the right internal thoracic artery without evidence of rib or sternal fractures, prompting TAE to stabilize circulation (Figure 1). Unfortunately, the patient died 22 days after CPR due to organ dysfunction caused by sepsis from the intra‐abdominal abscess.

## 2 Discussion

Rib fractures are the most common complication of CPR and have been reported to occur in 31.6% of cases [1]. Internal thoracic artery injury is a rare complication, occurring in 2.3% of cases, with a median CPR duration of 66 min [2]. In previously reported cases, antiplatelet agents were frequently administered because acute coronary syndrome was suspected, and anticoagulation therapy was initiated when VA‐ECMO was introduced [2, 3]. Mechanical circulatory support, targeted temperature management, and heparin use have been suggested as potential contributors to hemorrhagic complications [3]. However, in the present case, internal thoracic artery injury occurred even after short‐duration CPR with high‐quality chest compressions and without associated rib or sternal fractures. Furthermore, persistent hemodynamic instability developed despite the absence of these iatrogenic bleeding risk factors.

Given that hypovolemia due to bleeding from the left inferior gluteal artery was considered the primary cause of cardiac arrest, aggressive fluid resuscitation was required. However, since the arrest was caused by hemorrhage, the necessary volume resuscitation may have transiently stabilized physiology and delayed recognition of additional bleeding sources, including injury to the internal thoracic artery. This case suggests that additional bleeding sites should be considered when hemodynamic instability persists after return of spontaneous circulation despite apparent control of the primary source.

## Author Contributions

**Tatsuya Fujihara:** visualization, writing – original draft, writing – review and editing. **Akiyo Horiguchi:** writing – original draft, writing – review and editing. **Takahito Taniura:** writing – review and editing. **Tetsuro Nikai:** writing – review and editing.

## Funding

The authors have nothing to report.

## Ethics Statement

The authors have nothing to report.

## Consent

The authors confirm that written informed consent has been obtained from the involved patient's relative.

## Conflicts of Interest

The authors declare no conflicts of interest.

## Data Availability

The data supporting the findings of this study are not publicly available due to patient confidentiality and privacy considerations but are available from the corresponding author on reasonable request.
